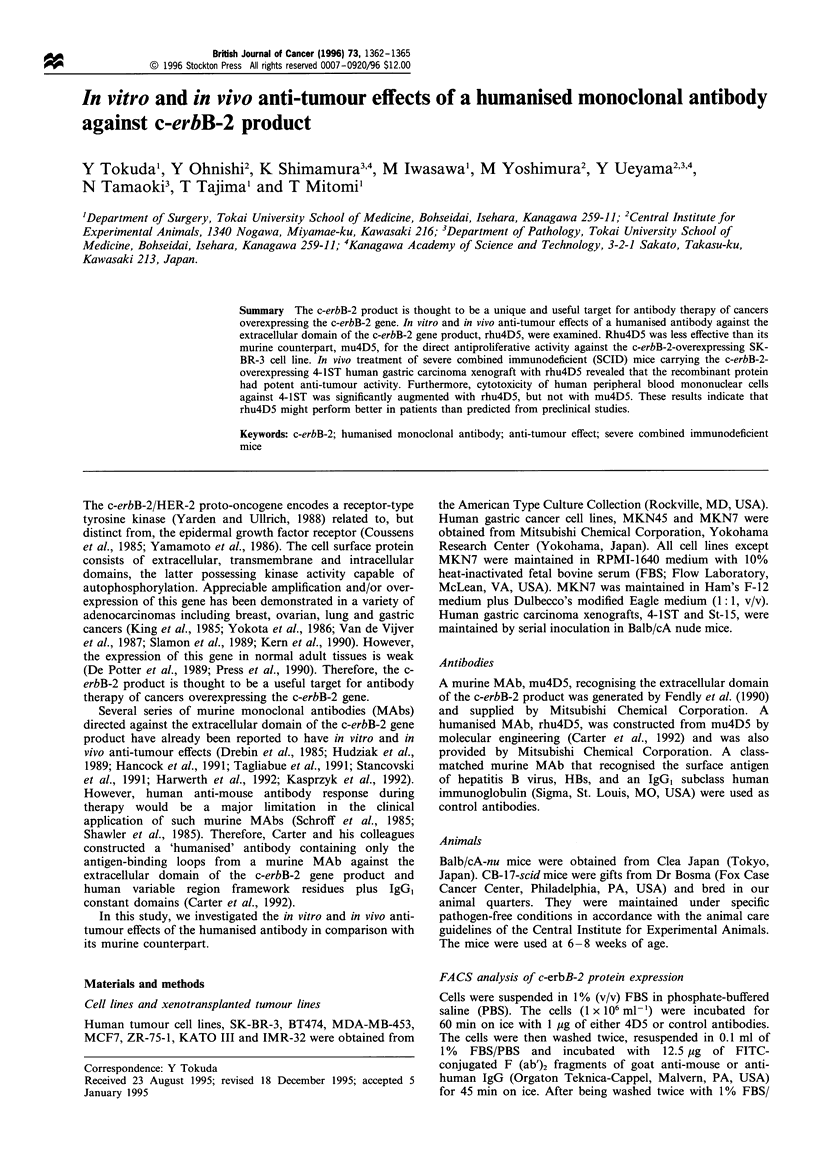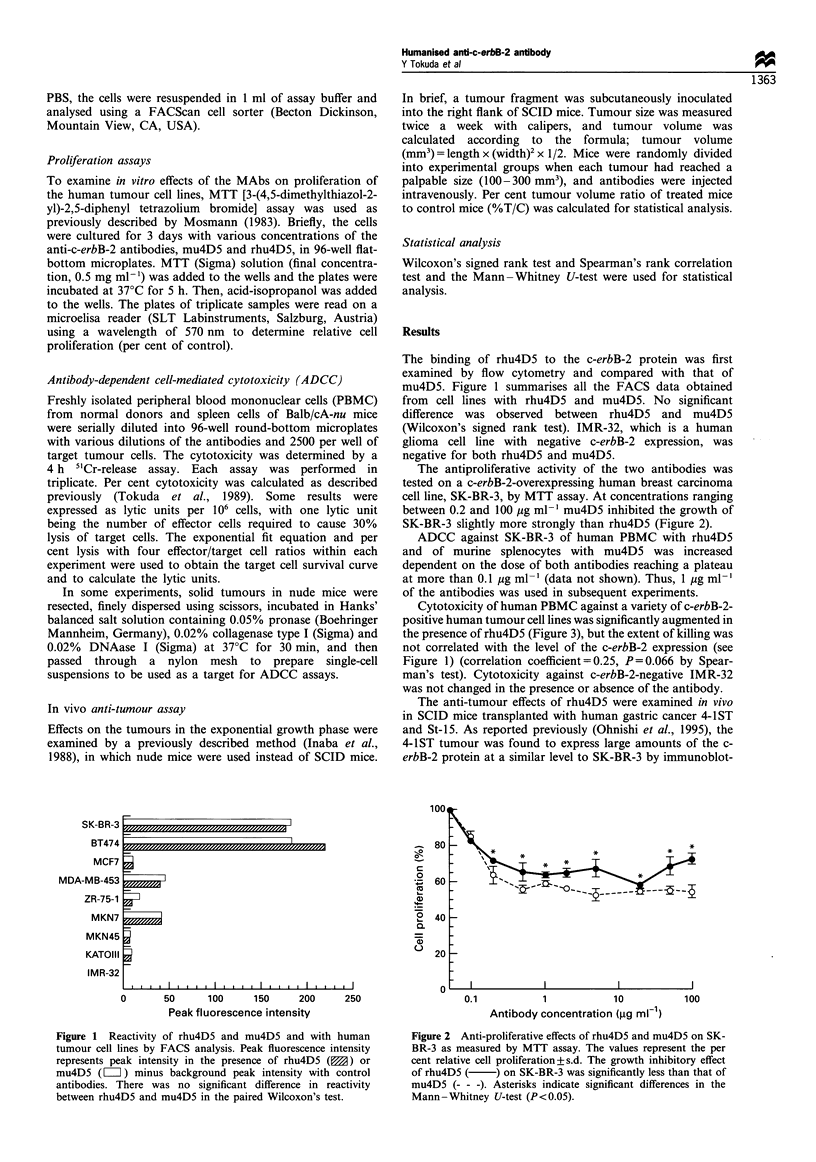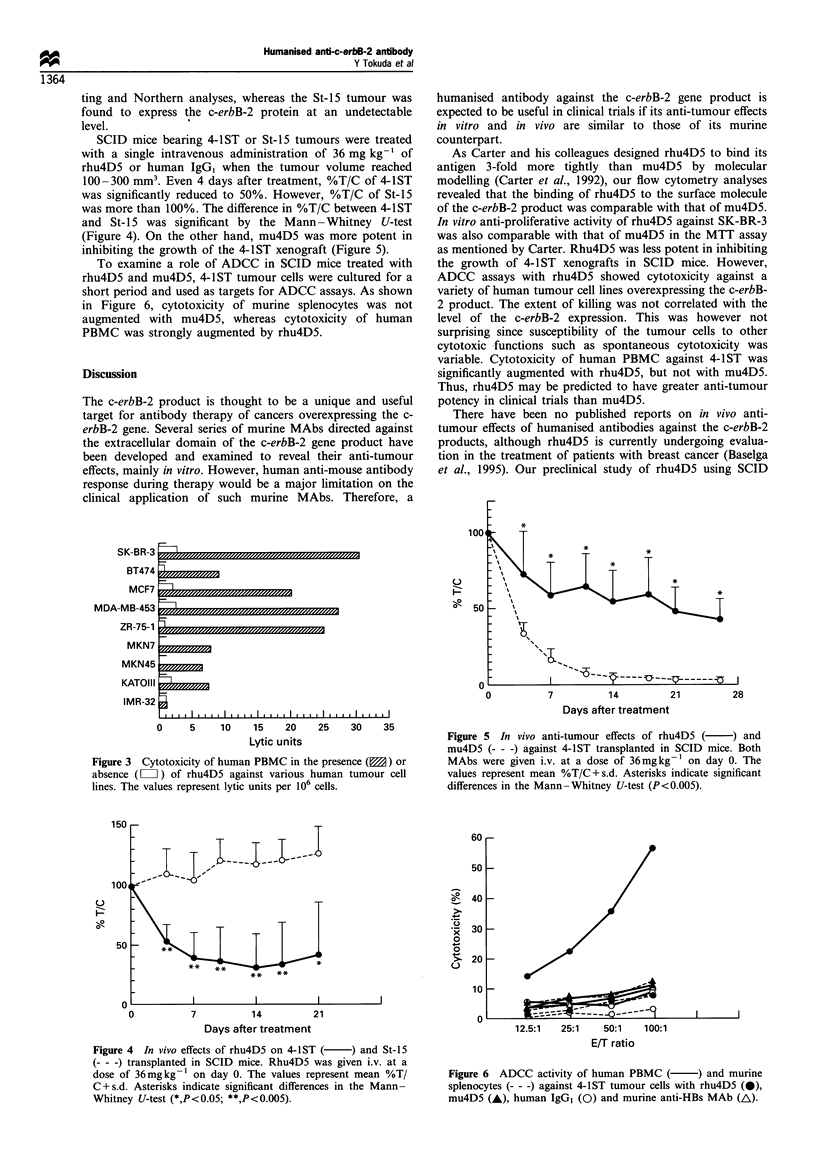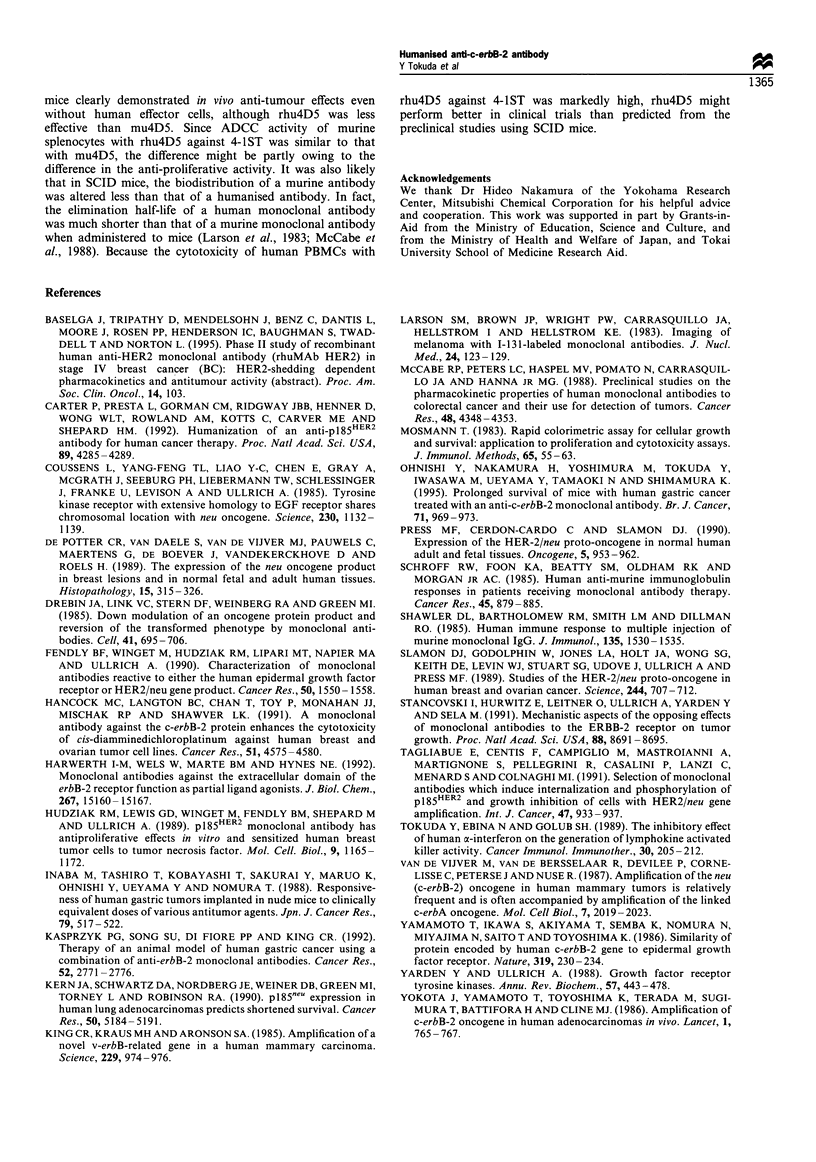# In vitro and in vivo anti-tumour effects of a humanised monoclonal antibody against c-erbB-2 product.

**DOI:** 10.1038/bjc.1996.259

**Published:** 1996-06

**Authors:** Y. Tokuda, Y. Ohnishi, K. Shimamura, M. Iwasawa, M. Yoshimura, Y. Ueyama, N. Tamaoki, T. Tajima, T. Mitomi

**Affiliations:** Department of Surgery, Tokai University School of Medicine, Kanagawa, Japan.

## Abstract

The c-erbB-2 product is thought to be a unique and useful target for antibody therapy of cancers overexpressing the c-erbB-2 gene. In vitro and in vivo anti-tumour effects of a humanised antibody against the extracellular domain of the c-erbB-2 gene product, rhu4D5, were examined. Rhu4D5 was less effective than its murine counterpart, mu4D5, for the direct antiproliferative activity against the c-erbB-2-overexpressing SK-BR-3 cell line. In vivo treatment of severe combined immunodeficient (SCID) mice carrying the c-erbB-2-overexpressing 4-1ST human gastric carcinoma xenograft with 4hu4D5 revealed that the recombinant protein had potent anti-tumour activity. Furthermore, cytotoxicity of human peripheral blood mononuclear cells against 4-1ST was significantly augmented with rhu4D5, but not with mu4D5. These results indicate that rhu4D5 might perform better in patients than predicted from preclinical studies.


British Journal of Cancer (1996) 73, 1362-1365
© 1996 Stockton Press All rights reserved 0007-0920/96 $12.00

### *In vitro* and *in vivo* anti-tumour effects of a humanised monoclonal antibody against c-*erb*B-2 product

Y Tokuda[1], Y Ohnishi[2], K Shimamura[3,4], M Iwasawa[1], M Yoshimura[2], Y Ueyama[2,3,4], N Tamaoki[3], T Tajima[1] and T Mitomi[1]

[1]*Department of Surgery, Tokai University School of Medicine, Bohseidai, Isehara, Kanagawa 259-11;* [2]*Central Institute for Experimental Animals, 1340 Nogawa, Miyamae-ku, Kawasaki 216;* [3]*Department of Pathology, Tokai University School of Medicine, Bohseidai, Isehara, Kanagawa 259-11;* [4]*Kanagawa Academy of Science and Technology, 3-2-1 Sakato, Takasu-ku, Kawasaki 213, Japan.*

**Summary** The c-*erb*B-2 product is thought to be a unique and useful target for antibody therapy of cancers overexpressing the c-*erb*B-2 gene. *In vitro* and *in vivo* anti-tumour effects of a humanised antibody against the extracellular domain of the c-*erb*B-2 gene product, rhu4D5, were examined. Rhu4D5 was less effective than its murine counterpart, mu4D5, for the direct antiproliferative activity against the c-*erb*B-2-overexpressing SK-BR-3 cell line. *In vivo* treatment of severe combined immunodeficient (SCID) mice carrying the c-*erb*B-2-overexpressing 4-1ST human gastric carcinoma xenograft with rhu4D5 revealed that the recombinant protein had potent anti-tumour activity. Furthermore, cytotoxicity of human peripheral blood mononuclear cells against 4-1ST was significantly augmented with rhu4D5, but not with mu4D5. These results indicate that rhu4D5 might perform better in patients than predicted from preclinical studies.

**Keywords:** c-*erb*B-2; humanised monoclonal antibody; anti-tumour effect; severe combined immunodeficient mice

The c-*erb*B-2/HER-2 proto-oncogene encodes a receptor-type tyrosine kinase (Yarden and Ullrich, 1988) related to, but distinct from, the epidermal growth factor receptor (Coussens *et al.*, 1985; Yamamoto *et al.*, 1986). The cell surface protein consists of extracellular, transmembrane and intracellular domains, the latter possessing kinase activity capable of autophosphorylation. Appreciable amplification and/or overexpression of this gene has been demonstrated in a variety of adenocarcinomas including breast, ovarian, lung and gastric cancers (King *et al.*, 1985; Yokota *et al.*, 1986; Van de Vijver *et al.*, 1987; Slamon *et al.*, 1989; Kern *et al.*, 1990). However, the expression of this gene in normal adult tissues is weak (De Potter *et al.*, 1989; Press *et al.*, 1990). Therefore, the c-*erb*B-2 product is thought to be a useful target for antibody therapy of cancers overexpressing the c-*erb*B-2 gene.

Several series of murine monoclonal antibodies (MAbs) directed against the extracellular domain of the c-*erb*B-2 gene product have already been reported to have *in vitro* and *in vivo* anti-tumour effects (Drebin *et al.*, 1985; Hudziak *et al.*, 1989; Hancock *et al.*, 1991; Tagliabue *et al.*, 1991; Stancovski *et al.*, 1991; Harwerth *et al.*, 1992; Kasprzyk *et al.*, 1992). However, human anti-mouse antibody response during therapy would be a major limitation in the clinical application of such murine MAbs (Schroff *et al.*, 1985; Shawler *et al.*, 1985). Therefore, Carter and his colleagues constructed a 'humanised' antibody containing only the antigen-binding loops from a murine MAb against the extracellular domain of the c-*erb*B-2 gene product and human variable region framework residues plus $IgG_1$ constant domains (Carter *et al.*, 1992).

In this study, we investigated the *in vitro* and *in vivo* anti-tumour effects of the humanised antibody in comparison with its murine counterpart.

### Materials and methods

### *Cell lines and xenotransplanted tumour lines*

Human tumour cell lines, SK-BR-3, BT474, MDA-MB-453, MCF7, ZR-75-1, KATO III and IMR-32 were obtained from the American Type Culture Collection (Rockville, MD, USA). Human gastric cancer cell lines, MKN45 and MKN7 were obtained from Mitsubishi Chemical Corporation, Yokohama Research Center (Yokohama, Japan). All cell lines except MKN7 were maintained in RPMI-1640 medium with 10% heat-inactivated fetal bovine serum (FBS; Flow Laboratory, McLean, VA, USA). MKN7 was maintained in Ham's F-12 medium plus Dulbecco's modified Eagle medium (1:1, v/v). Human gastric carcinoma xenografts, 4-1ST and St-15, were maintained by serial inoculation in Balb/cA nude mice.

### *Antibodies*

A murine MAb, mu4D5, recognising the extracellular domain of the c-*erb*B-2 product was generated by Fendly *et al.* (1990) and supplied by Mitsubishi Chemical Corporation. A humanised MAb, rhu4D5, was constructed from mu4D5 by molecular engineering (Carter *et al.*, 1992) and was also provided by Mitsubishi Chemical Corporation. A class-matched murine MAb that recognised the surface antigen of hepatitis B virus, HBs, and an $IgG_1$ subclass human immunoglobulin (Sigma, St. Louis, MO, USA) were used as control antibodies.

### *Animals*

Balb/cA-*nu* mice were obtained from Clea Japan (Tokyo, Japan). CB-17-*scid* mice were gifts from Dr Bosma (Fox Case Cancer Center, Philadelphia, PA, USA) and bred in our animal quarters. They were maintained under specific pathogen-free conditions in accordance with the animal care guidelines of the Central Institute for Experimental Animals. The mice were used at 6–8 weeks of age.

### *FACS analysis of c-*erbB*-2 protein expression*

Cells were suspended in 1% (v/v) FBS in phosphate-buffered saline (PBS). The cells ($1 \times 10^6$ $ml^{-1}$) were incubated for 60 min on ice with 1 $\mu g$ of either 4D5 or control antibodies. The cells were then washed twice, resuspended in 0.1 ml of 1% FBS/PBS and incubated with 12.5 $\mu g$ of FITC-conjugated F $(ab')_2$ fragments of goat anti-mouse or anti-human IgG (Orgaton Teknica-Cappel, Malvern, PA, USA) for 45 min on ice. After being washed twice with 1% FBS/

Correspondence: Y Tokuda
Received 23 August 1995; revised 18 December 1995; accepted 5 January 1995

PBS, the cells were resuspended in 1 ml of assay buffer and analysed using a FACScan cell sorter (Becton Dickinson, Mountain View, CA, USA).

*Proliferation assays*

To examine *in vitro* effects of the MAbs on proliferation of the human tumour cell lines, MTT [3-(4,5-dimethylthiazol-2-yl)-2,5-diphenyl tetrazolium bromide] assay was used as previously described by Mosmann (1983). Briefly, the cells were cultured for 3 days with various concentrations of the anti-c-*erb*B-2 antibodies, mu4D5 and rhu4D5, in 96-well flat-bottom microplates. MTT (Sigma) solution (final concentration, 0.5 mg $ml^{-1}$) was added to the wells and the plates were incubated at 37°C for 5 h. Then, acid-isopropanol was added to the wells. The plates of triplicate samples were read on a microelisa reader (SLT Labinstruments, Salzburg, Austria) using a wavelength of 570 nm to determine relative cell proliferation (per cent of control).

*Antibody-dependent cell-mediated cytotoxicity (ADCC)*

Freshly isolated peripheral blood mononuclear cells (PBMC) from normal donors and spleen cells of Balb/cA-*nu* mice were serially diluted into 96-well round-bottom microplates with various dilutions of the antibodies and 2500 per well of target tumour cells. The cytotoxicity was determined by a 4 h $^{51}$Cr-release assay. Each assay was performed in triplicate. Per cent cytotoxicity was calculated as described previously (Tokuda *et al.*, 1989). Some results were expressed as lytic units per $10^6$ cells, with one lytic unit being the number of effector cells required to cause 30% lysis of target cells. The exponential fit equation and per cent lysis with four effector/target cell ratios within each experiment were used to obtain the target cell survival curve and to calculate the lytic units.

In some experiments, solid tumours in nude mice were resected, finely dispersed using scissors, incubated in Hanks' balanced salt solution containing 0.05% pronase (Boehringer Mannheim, Germany), 0.02% collagenase type I (Sigma) and 0.02% DNAase I (Sigma) at 37°C for 30 min, and then passed through a nylon mesh to prepare single-cell suspensions to be used as a target for ADCC assays.

In vivo *anti-tumour assay*

Effects on the tumours in the exponential growth phase were examined by a previously described method (Inaba *et al.*, 1988), in which nude mice were used instead of SCID mice. In brief, a tumour fragment was subcutaneously inoculated into the right flank of SCID mice. Tumour size was measured twice a week with calipers, and tumour volume was calculated according to the formula; tumour volume ($mm^3$) = length × (width)$^2$ × 1/2. Mice were randomly divided into experimental groups when each tumour had reached a palpable size (100–300 $mm^3$), and antibodies were injected intravenously. Per cent tumour volume ratio of treated mice to control mice (%T/C) was calculated for statistical analysis.

*Statistical analysis*

Wilcoxon's signed rank test and Spearman's rank correlation test and the Mann–Whitney *U*-test were used for statistical analysis.

### Results

The binding of rhu4D5 to the c-*erb*B-2 protein was first examined by flow cytometry and compared with that of mu4D5. Figure 1 summarises all the FACS data obtained from cell lines with rhu4D5 and mu4D5. No significant difference was observed between rhu4D5 and mu4D5 (Wilcoxon's signed rank test). IMR-32, which is a human glioma cell line with negative c-*erb*B-2 expression, was negative for both rhu4D5 and mu4D5.

The antiproliferative activity of the two antibodies was tested on a c-*erb*B-2-overexpressing human breast carcinoma cell line, SK-BR-3, by MTT assay. At concentrations ranging between 0.2 and 100 $\mu$g $ml^{-1}$ mu4D5 inhibited the growth of SK-BR-3 slightly more strongly than rhu4D5 (Figure 2).

ADCC against SK-BR-3 of human PBMC with rhu4D5 and of murine splenocytes with mu4D5 was increased dependent on the dose of both antibodies reaching a plateau at more than 0.1 $\mu$g $ml^{-1}$ (data not shown). Thus, 1 $\mu$g $ml^{-1}$ of the antibodies was used in subsequent experiments.

Cytotoxicity of human PBMC against a variety of c-*erb*B-2-positive human tumour cell lines was significantly augmented in the presence of rhu4D5 (Figure 3), but the extent of killing was not correlated with the level of the c-*erb*B-2 expression (see Figure 1) (correlation coefficient = 0.25, $P$ = 0.066 by Spearman's test). Cytotoxicity against c-*erb*B-2-negative IMR-32 was not changed in the presence or absence of the antibody.

The anti-tumour effects of rhu4D5 were examined *in vivo* in SCID mice transplanted with human gastric cancer 4-1ST and St-15. As reported previously (Ohnishi *et al.*, 1995), the 4-1ST tumour was found to express large amounts of the c-*erb*B-2 protein at a similar level to SK-BR-3 by immunoblot-

**Figure 1** Reactivity of rhu4D5 and mu4D5 and with human tumour cell lines by FACS analysis. Peak fluorescence intensity represents peak intensity in the presence of rhu4D5 (hatched bar) or mu4D5 (open bar) minus background peak intensity with control antibodies. There was no significant difference in reactivity between rhu4D5 and mu4D5 in the paired Wilcoxon's test.

**Figure 2** Anti-proliferative effects of rhu4D5 and mu4D5 on SK-BR-3 as measured by MTT assay. The values represent the per cent relative cell proliferation ± s.d. The growth inhibitory effect of rhu4D5 (——) on SK-BR-3 was significantly less than that of mu4D5 (- - -). Asterisks indicate significant differences in the Mann–Whitney *U*-test ($P$ < 0.05).

ting and Northern analyses, whereas the St-15 tumour was found to express the c-*erb*B-2 protein at an undetectable level.

SCID mice bearing 4-1ST or St-15 tumours were treated with a single intravenous administration of 36 mg $kg^{-1}$ of rhu4D5 or human $IgG_1$ when the tumour volume reached 100–300 $mm^3$. Even 4 days after treatment, %T/C of 4-1ST was significantly reduced to 50%. However, %T/C of St-15 was more than 100%. The difference in %T/C between 4-1ST and St-15 was significant by the Mann–Whitney *U*-test (Figure 4). On the other hand, mu4D5 was more potent in inhibiting the growth of the 4-1ST xenograft (Figure 5).

To examine a role of ADCC in SCID mice treated with rhu4D5 and mu4D5, 4-1ST tumour cells were cultured for a short period and used as targets for ADCC assays. As shown in Figure 6, cytotoxicity of murine splenocytes was not augmented with mu4D5, whereas cytotoxicity of human PBMC was strongly augmented by rhu4D5.

### Discussion

The c-*erb*B-2 product is thought to be a unique and useful target for antibody therapy of cancers overexpressing the c-*erb*B-2 gene. Several series of murine MAbs directed against the extracellular domain of the c-*erb*B-2 gene product have been developed and examined to reveal their anti-tumour effects, mainly *in vitro*. However, human anti-mouse antibody response during therapy would be a major limitation on the clinical application of such murine MAbs. Therefore, a humanised antibody against the c-*erb*B-2 gene product is expected to be useful in clinical trials if its anti-tumour effects *in vitro* and *in vivo* are similar to those of its murine counterpart.

As Carter and his colleagues designed rhu4D5 to bind its antigen 3-fold more tightly than mu4D5 by molecular modelling (Carter *et al.*, 1992), our flow cytometry analyses revealed that the binding of rhu4D5 to the surface molecule of the c-*erb*B-2 product was comparable with that of mu4D5. *In vitro* anti-proliferative activity of rhu4D5 against SK-BR-3 was also comparable with that of mu4D5 in the MTT assay as mentioned by Carter. Rhu4D5 was less potent in inhibiting the growth of 4-1ST xenografts in SCID mice. However, ADCC assays with rhu4D5 showed cytotoxicity against a variety of human tumour cell lines overexpressing the c-*erb*B-2 product. The extent of killing was not correlated with the level of the c-*erb*B-2 expression. This was however not surprising since susceptibility of the tumour cells to other cytotoxic functions such as spontaneous cytotoxicity was variable. Cytotoxicity of human PBMC against 4-1ST was significantly augmented with rhu4D5, but not with mu4D5. Thus, rhu4D5 may be predicted to have greater anti-tumour potency in clinical trials than mu4D5.

There have been no published reports on *in vivo* anti-tumour effects of humanised antibodies against the c-*erb*B-2 products, although rhu4D5 is currently undergoing evaluation in the treatment of patients with breast cancer (Baselga *et al.*, 1995). Our preclinical study of rhu4D5 using SCID

**Figure 3** Cytotoxicity of human PBMC in the presence (▨) or absence (□) of rhu4D5 against various human tumour cell lines. The values represent lytic units per $10^6$ cells.

**Figure 4** *In vivo* effects of rhu4D5 on 4-1ST (——) and St-15 (- - -) transplanted in SCID mice. Rhu4D5 was given i.v. at a dose of 36 mg $kg^{-1}$ on day 0. The values represent mean %T/C + s.d. Asterisks indicate significant differences in the Mann–Whitney *U*-test (*, $P<0.05$; **, $P<0.005$).

**Figure 5** *In vivo* anti-tumour effects of rhu4D5 (——) and mu4D5 (- - -) against 4-1ST transplanted in SCID mice. Both MAbs were given i.v. at a dose of 36 mg $kg^{-1}$ on day 0. The values represent mean %T/C + s.d. Asterisks indicate significant differences in the Mann–Whitney *U*-test ($P<0.005$).

**Figure 6** ADCC activity of human PBMC (——) and murine splenocytes (- - -) against 4-1ST tumour cells with rhu4D5 (●), mu4D5 (▲), human $IgG_1$ (○) and murine anti-HBs MAb (△).

mice clearly demonstrated *in vivo* anti-tumour effects even without human effector cells, although rhu4D5 was less effective than mu4D5. Since ADCC activity of murine splenocytes with rhu4D5 against 4-1ST was similar to that with mu4D5, the difference might be partly owing to the difference in the anti-proliferative activity. It was also likely that in SCID mice, the biodistribution of a murine antibody was altered less than that of a humanised antibody. In fact, the elimination half-life of a human monoclonal antibody was much shorter than that of a murine monoclonal antibody when administered to mice (Larson *et al*., 1983; McCabe *et al*., 1988). Because the cytotoxicity of human PBMCs with rhu4D5 against 4-1ST was markedly high, rhu4D5 might perform better in clinical trials than predicted from the preclinical studies using SCID mice.

**Acknowledgements**
We thank Dr Hideo Nakamura of the Yokohama Research Center, Mitsubishi Chemical Corporation for his helpful advice and cooperation. This work was supported in part by Grants-in-Aid from the Ministry of Education, Science and Culture, and from the Ministry of Health and Welfare of Japan, and Tokai University School of Medicine Research Aid.

### References

BASELGA J, TRIPATHY D, MENDELSOHN J, BENZ C, DANTIS L, MOORE J, ROSEN PP, HENDERSON IC, BAUGHMAN S, TWADDELL T AND NORTON L. (1995). Phase II study of recombinant human anti-HER2 monoclonal antibody (rhuMAb HER2) in stage IV breast cancer (BC): HER2-shedding dependent pharmacokinetics and antitumour activity (abstract). *Proc. Am. Soc. Clin. Oncol.*, **14**, 103.

CARTER P, PRESTA L, GORMAN CM, RIDGWAY JBB, HENNER D, WONG WLT, ROWLAND AM, KOTTS C, CARVER ME AND SHEPARD HM. (1992). Humanization of an anti-p185$^{HER2}$ antibody for human cancer therapy. *Proc. Natl Acad. Sci. USA*, **89**, 4285–4289.

COUSSENS L, YANG-FENG TL, LIAO Y-C, CHEN E, GRAY A, MCGRATH J, SEEBURG PH, LIEBERMANN TW, SCHLESSINGER J, FRANKE U, LEVISON A AND ULLRICH A. (1985). Tyrosine kinase receptor with extensive homology to EGF receptor shares chromosomal location with *neu* oncogene. *Science*, **230**, 1132–1139.

DE POTTER CR, VAN DAELE S, VAN DE VIJVER MJ, PAUWELS C, MAERTENS G, DE BOEVER J, VANDEKERCKHOVE D AND ROELS H. (1989). The expression of the *neu* oncogene product in breast lesions and in normal fetal and adult human tissues. *Histopathology*, **15**, 315–326.

DREBIN JA, LINK VC, STERN DF, WEINBERG RA AND GREEN MI. (1985). Down modulation of an oncogene protein product and reversion of the transformed phenotype by monoclonal antibodies. *Cell*, **41**, 695–706.

FENDLY BF, WINGET M, HUDZIAK RM, LIPARI MT, NAPIER MA AND ULLRICH A. (1990). Characterization of monoclonal antibodies reactive to either the human epidermal growth factor receptor or HER2/neu gene product. *Cancer Res.*, **50**, 1550–1558.

HANCOCK MC, LANGTON BC, CHAN T, TOY P, MONAHAN JJ, MISCHAK RP AND SHAWVER LK. (1991). A monoclonal antibody against the c-*erb*B-2 protein enhances the cytotoxicity of *cis*-diamminedichloroplatinum against human breast and ovarian tumor cell lines. *Cancer Res.*, **51**, 4575–4580.

HARWERTH I-M, WELS W, MARTE BM AND HYNES NE. (1992). Monoclonal antibodies against the extracellular domain of the *erb*B-2 receptor function as partial ligand agonists. *J. Biol. Chem.*, **267**, 15160–15167.

HUDZIAK RM, LEWIS GD, WINGET M, FENDLY BM, SHEPARD M AND ULLRICH A. (1989). p185$^{HER2}$ monoclonal antibody has antiproliferative effects *in vitro* and sensitized human breast tumor cells to tumor necrosis factor. *Mol. Cell. Biol.*, **9**, 1165–1172.

INABA M, TASHIRO T, KOBAYASHI T, SAKURAI Y, MARUO K, OHNISHI Y, UEYAMA Y AND NOMURA T. (1988). Responsiveness of human gastric tumors implanted in nude mice to clinically equivalent doses of various antitumor agents. *Jpn. J. Cancer Res.*, **79**, 517–522.

KASPRZYK PG, SONG SU, DI FIORE PP AND KING CR. (1992). Therapy of an animal model of human gastric cancer using a combination of anti-*erb*B-2 monoclonal antibodies. *Cancer Res.*, **52**, 2771–2776.

KERN JA, SCHWARTZ DA, NORDBERG JE, WEINER DB, GREEN MI, TORNEY L AND ROBINSON RA. (1990). p185$^{neu}$ expression in human lung adenocarcinomas predicts shortened survival. *Cancer Res.*, **50**, 5184–5191.

KING CR, KRAUS MH AND ARONSON SA. (1985). Amplification of a novel v-*erb*B-related gene in a human mammary carcinoma. *Science*, **229**, 974–976.

LARSON SM, BROWN JP, WRIGHT PW, CARRASQUILLO JA, HELLSTROM I AND HELLSTROM KE. (1983). Imaging of melanoma with I-131-labeled monoclonal antibodies. *J. Nucl. Med.*, **24**, 123–129.

MCCABE RP, PETERS LC, HASPEL MV, POMATO N, CARRASQUILLO JA AND HANNA JR MG. (1988). Preclinical studies on the pharmacokinetic properties of human monoclonal antibodies to colorectal cancer and their use for detection of tumors. *Cancer Res.*, **48**, 4348–4353.

MOSMANN T. (1983). Rapid colorimetric assay for cellular growth and survival: application to proliferation and cytotoxicity assays. *J. Immunol. Methods*, **65**, 55–63.

OHNISHI Y, NAKAMURA H, YOSHIMURA M, TOKUDA Y, IWASAWA M, UEYAMA Y, TAMAOKI N AND SHIMAMURA K. (1995). Prolonged survival of mice with human gastric cancer treated with an anti-c-*erb*B-2 monoclonal antibody. *Br. J. Cancer*, **71**, 969–973.

PRESS MF, CERDON-CARDO C AND SLAMON DJ. (1990). Expression of the HER-2/*neu* proto-oncogene in normal human adult and fetal tissues. *Oncogene*, **5**, 953–962.

SCHROFF RW, FOON KA, BEATTY SM, OLDHAM RK AND MORGAN JR AC. (1985). Human anti-murine immunoglobulin responses in patients receiving monoclonal antibody therapy. *Cancer Res.*, **45**, 879–885.

SHAWLER DL, BARTHOLOMEW RM, SMITH LM AND DILLMAN RO. (1985). Human immune response to multiple injection of murine monoclonal IgG. *J. Immunol.*, **135**, 1530–1535.

SLAMON DJ, GODOLPHIN W, JONES LA, HOLT JA, WONG SG, KEITH DE, LEVIN WJ, STUART SG, UDOVE J, ULLRICH A AND PRESS MF. (1989). Studies of the HER-2/*neu* proto-oncogene in human breast and ovarian cancer. *Science*, **244**, 707–712.

STANCOVSKI I, HURWITZ E, LEITNER O, ULLRICH A, YARDEN Y AND SELA M. (1991). Mechanistic aspects of the opposing effects of monoclonal antibodies to the ERBB-2 receptor on tumor growth. *Proc. Natl Acad. Sci. USA*, **88**, 8691–8695.

TAGLIABUE E, CENTIS F, CAMPIGLIO M, MASTROIANNI A, MARTIGNONE S, PELLEGRINI R, CASALINI P, LANZI C, MENARD S AND COLNAGHI MI. (1991). Selection of monoclonal antibodies which induce internalization and phosphorylation of p185$^{HER2}$ and growth inhibition of cells with HER2/*neu* gene amplification. *Int. J. Cancer*, **47**, 933–937.

TOKUDA Y, EBINA N AND GOLUB SH. (1989). The inhibitory effect of human $\alpha$-interferon on the generation of lymphokine activated killer activity. *Cancer Immunol. Immunother.*, **30**, 205–212.

VAN DE VIJVER M, VAN DE BERSSELAAR R, DEVILEE P, CORNELISSE C, PETERSE J AND NUSE R. (1987). Amplification of the *neu* (c-*erb*B-2) oncogene in human mammary tumors is relatively frequent and is often accompanied by amplification of the linked c-*erb*A oncogene. *Mol. Cell Biol.*, **7**, 2019–2023.

YAMAMOTO T, IKAWA S, AKIYAMA T, SEMBA K, NOMURA N, MIYAJIMA N, SAITO T AND TOYOSHIMA K. (1986). Similarity of protein encoded by human c-*erb*B-2 gene to epidermal growth factor receptor. *Nature*, **319**, 230–234.

YARDEN Y AND ULLRICH A. (1988). Growth factor receptor tyrosine kinases. *Annu. Rev. Biochem.*, **57**, 443–478.

YOKOTA J, YAMAMOTO T, TOYOSHIMA K, TERADA M, SUGIMURA T, BATTIFORA H AND CLINE MJ. (1986). Amplification of c-*erb*B-2 oncogene in human adenocarcinomas *in vivo*. *Lancet*, **1**, 765–767.